# Supplementary material for: Defining the Protease and Protease Inhibitor (P/PI) Proteomes of Healthy and Diseased Human Skin by Modified Systematic Review
Source: Biomolecules. 2022 Mar 20;12(3):475. doi: 10.3390/biom12030475 (PMC8946613; doi:10.3390/biom12030475)
Supplement: Supplementary file 1 [file biomolecules-12-00475-s001.zip › biomolecules-1602100-SI/Supplementary Figures 1-2_V2.pdf]

Supplementary Figure S1. Number of published articles for each protease

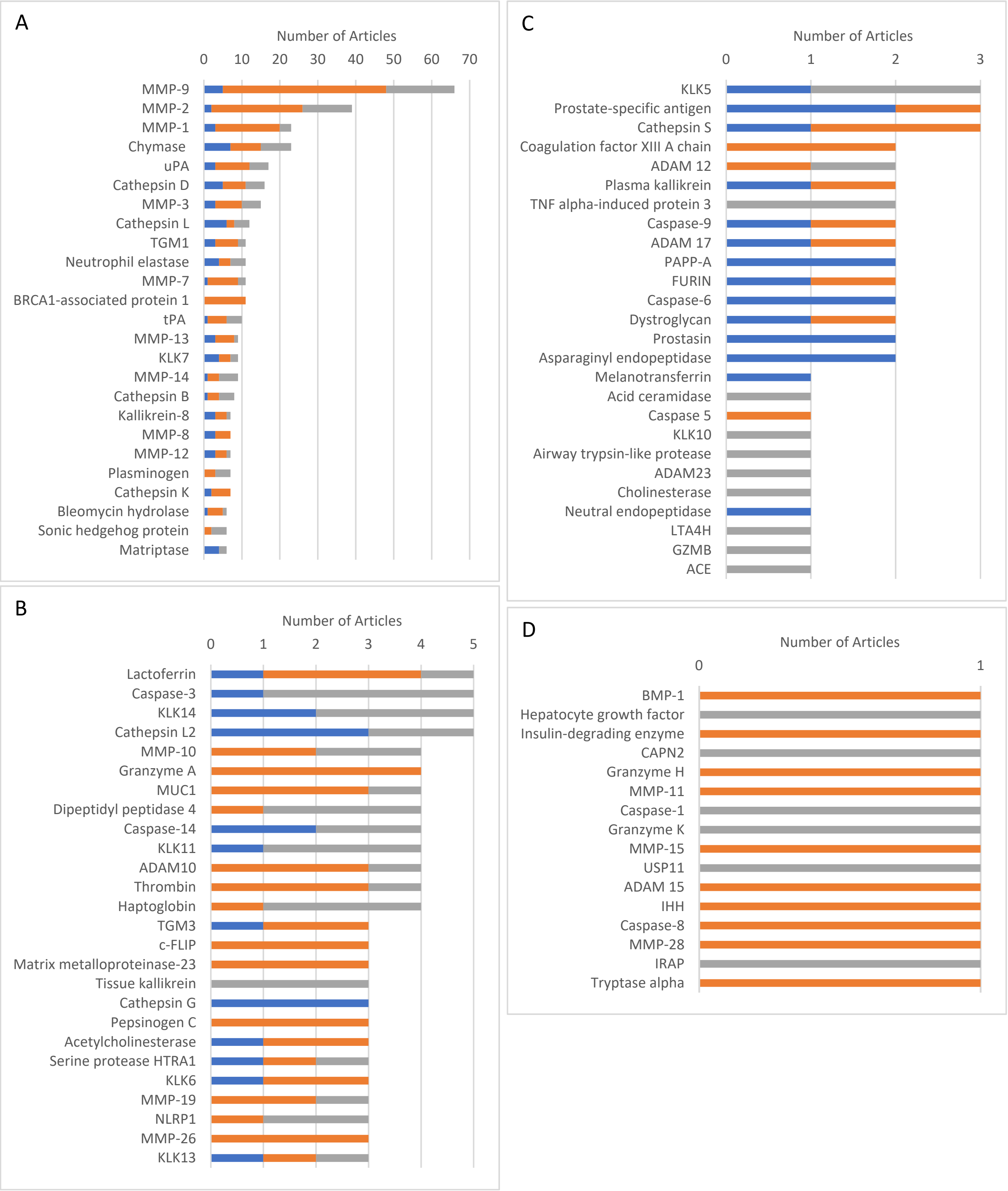

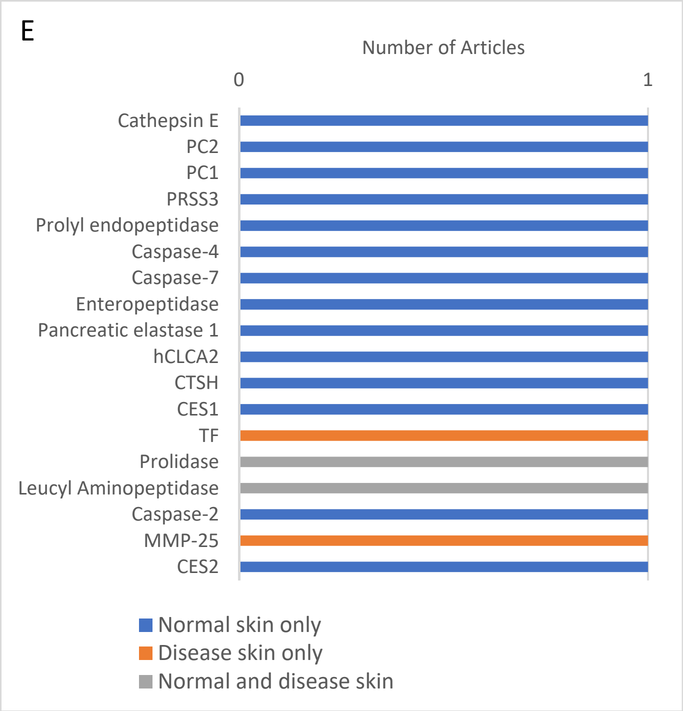
E
Number of Articles
0
1
Cathepsin E
PC2
PC1
PRSS3
Prolyl endopeptidase
Caspase-4
Caspase-7
Enteropeptidase
Pancreatic elastase 1
hCLCA2
CTSH
CES1
TF
Prolidase
Leucyl Aminopeptidase
Caspase-2
MMP-25
CES2
Normal skin only
Disease skin only
Normal and disease skin

Supplementary Figure S2. Number of published articles for each protease inhibitor

A

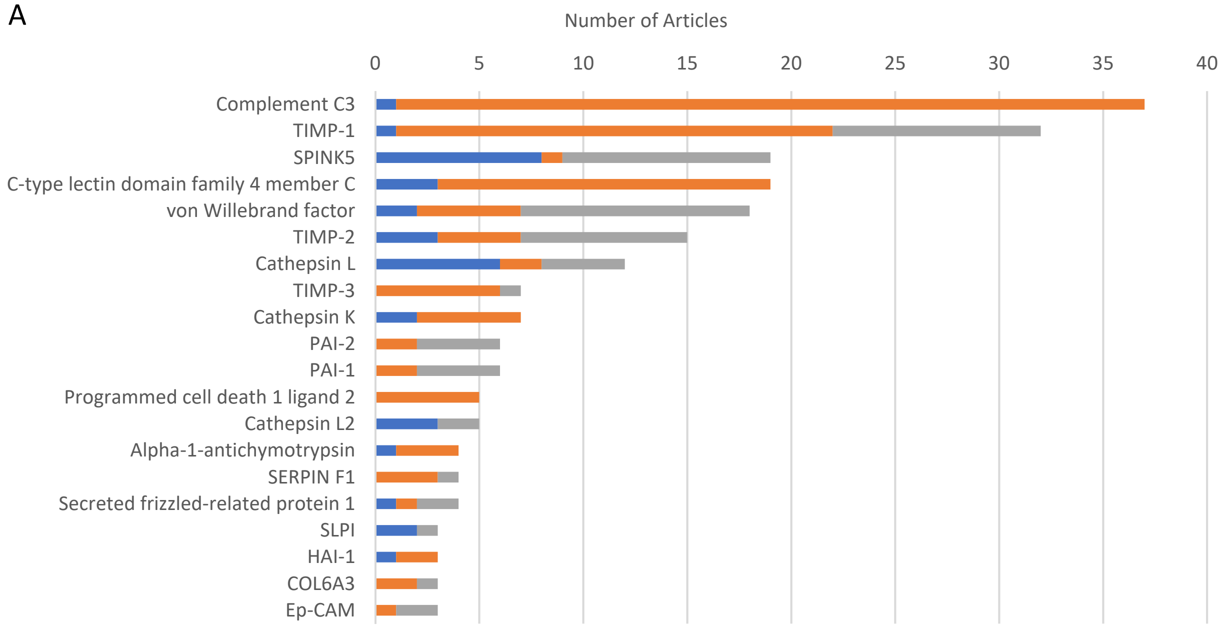

B

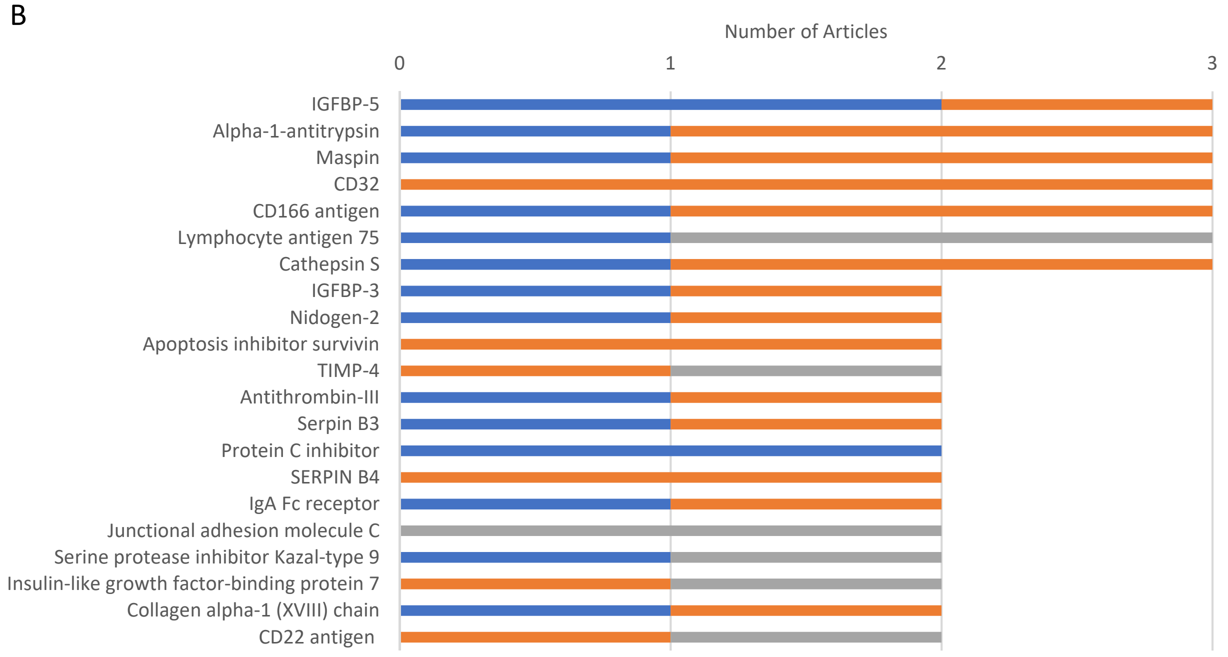

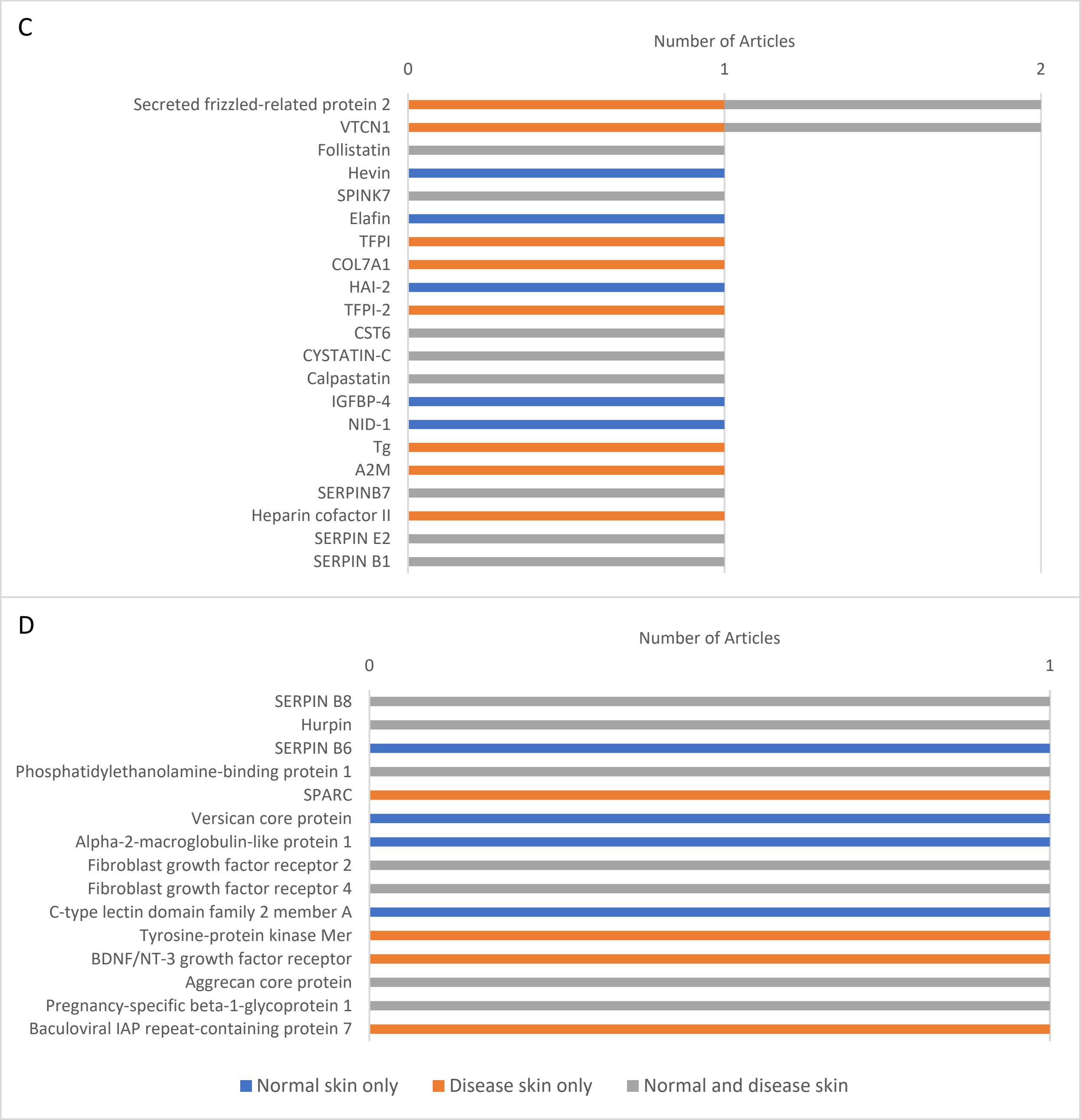
C
Number of Articles
0
1
2
Secreted frizzled-related protein 2
VTCN1
Follistatin
Hevin
SPINK7
Elafin
TFPI
COL7A1
HAI-2
TFPI-2
CST6
CYSTATIN-C
Calpastatin
IGFBP-4
NID-1
Tg
A2M
SERPINB7
Heparin cofactor II
SERPIN E2
SERPIN B1
D
Number of Articles
0
1
SERPIN B8
Hurpin
SERPIN B6
Phosphatidylethanolamine-binding protein 1
SPARC
Versican core protein
Alpha-2-macroglobulin-like protein 1
Fibroblast growth factor receptor 2
Fibroblast growth factor receptor 4
C-type lectin domain family 2 member A
Tyrosine-protein kinase Mer
BDNF/NT-3 growth factor receptor
Aggrecan core protein
Pregnancy-specific beta-1-glycoprotein 1
Baculoviral IAP repeat-containing protein 7
Normal skin only
Disease skin only
Normal and disease skin
